# Phenotypic and genetic variability of a tetraploid wheat collection grown in Kazakhstan

**DOI:** 10.18699/VJ20.654

**Published:** 2020-10

**Authors:** A. Zatybekov, S. Anuarbek, S. Abugalieva, Y. Turuspekov

**Affiliations:** Institute of Plant Biology and Biotechnology, Almaty, Kazakhstan; Institute of Plant Biology and Biotechnology, Almaty, Kazakhstan Al-Farabi Kazakh National University, Almaty, Kazakhstan; Institute of Plant Biology and Biotechnology, Almaty, Kazakhstan; Institute of Plant Biology and Biotechnology, Almaty, Kazakhstan Al-Farabi Kazakh National University, Almaty, Kazakhstan

**Keywords:** Triticum turgidum, genetic diversity, SSR markers, agronomic traits, Triticum turgidum, генетическое разнообразие, SSR-маркеры, хозяйственно ценные признаки

## Abstract

New cultivars adapted to major durum wheat growing environments are essential for the cultivation of this crop. The development of new cultivars has required the availability of diverse genetic material and their extensive field trials. In this work, a collection of tetraploid wheat consisting of 85 accessions was tested in the field conditions of Almaty region during 2018 and 2019. The accessions were ranged according to nine agronomic traits studied, and accessions with the highest yield performance for Almaty region of Kazakhstan were revealed. The ANOVA suggested that the performance of agronomic traits were influenced both by Environment and Genotype. Also, the collection was analyzed using seven SSR (simple sequence repeats) markers. From 3 to 6 alleles per locus were revealed, with an average of 4.6, while the effective number of alleles was 2.8. Nei’s genetic diversity was in the range of 0.45–0.69. The results showed high values of polymorphism index content (PIC) in the range of 0.46–0.70, with an average of 0.62, suggesting that 6 out of 7 SSRs were highly informative (PIC > 0.5). Phylogenetic analysis of the collection has allowed the separation of accessions into six clusters. The local accessions were presented in all six clusters with the majority of them grouped in the first three clusters designated as A, B, and C, respectively. The relations between SSR markers and agronomic traits in the collection were studied. The results can be efficiently used for the enhancement of local breeding projects for the improvement of yield productivity in durum wheat.

## Abstract (ru)

В основе эффективных технологий выращивания полевых культур лежат новые сорта, адаптиро- ванные к условиям зоны производства. Создание таких сортов предполагает наличие коллекции с широким генетическим разнообразием и тщательные полевые экологические испытания. В данной работе коллекция тетраплоидной пшеницы, состоящая из 85 сортов и линий различного происхождения, была изучена в по- левых условиях Алматинской области Казахстана в 2018 и 2019 гг. Образцы коллекции были ранжированы по девяти изученным сельскохозяйственным признакам, в результате чего выявлены линии с высокой про- дуктивностью в условиях Алматинской области. С помощью дисперсионного анализа удалось установить, что как окружающая среда, так и генотип оказывают статистически высокое влияние на хозяйственно цен- ные признаки. Коллекция была исследована также с использованием семи микросателлитных SSR (simple sequence repeats) маркеров. Обнаружено от 3 до 6 аллелей на локус со средним значением 4.6, тогда как среднее значение эффективного числа аллелей равнялось 2.8. Индекс генетического разнообразия коллек- ции по Нею был высоким, в пределах 0.45–0.69. Значения PIC (polymorphism index content) варьировали от 0.46 до 0.70, при этом шесть из семи SSR показали высокую информативность (PIC > 0.5). Филогенетический анализ коллекции позволил разделить образцы на шесть кластеров. Местные образцы были представлены во всех шести кластерах; большинство из них было сгруппировано в первых трех кластерах, обозначенных как A, B и C. Изучена связь между определенными SSR-маркерами и агрономическими признаками в рас- сматриваемой коллекции. Полученные результаты могут быть эффективно использованы для усиления отечественных селекционных проектов для улучшения продуктивности твердой пшеницы.

## Introduction

Durum wheat (Triticum turgidum L. ssp. turgidum convar. durum (Desf.) MacKey) is a tetraploid species of wheat and is the main crop to producers of pasta and cereals. The growing area under durum wheat is about 17 million hectares in the world and production is 37 million tons (Kabbaj et al., 2017; Zaïm et al., 2017). In 2019, durum wheat production in Kazakhstan amounted to 560 thousand tons (https://agbz.kz). Other tetraploid wheat species Triticum turgidum L. ssp. turanicum (Jakubz.) Á. Löve & D. Löve, Triticum turgidum L. ssp. polonicum (L.) Thell., Triticum turgidum L. ssp. carthlicum (Nevski) Á. Löve & D. Löve, Triticum turgidum ssp. dicoc-cum (Shrank ex Schübler) Thell. are used as food and feed crops in different world regions. Wild species Triticum turgidum ssp. dicoccoides (Korn. ex Asch. & Graebn.) Thell. is also often included in crossing schemes as a source for resistance to abiotic and biotic stresses (De Vita, Taranto, 2019; Mujeeb-Kazi et al., 2019).

The enhancement of a breeding program largely depends on an understanding of adaptation-related patterns that affect the productivity of cereal crops, including durum wheat. One of the ways to study these patterns is the assessment of diverse germplasm collections, including relative wild and cultivated species and landraces, in a particular environmental condition, and evaluate genotype × environment interaction features (Anuarbek et al., 2020). Hence, the comprehensive study of the diverse germplasm is a very important prerequisite for the successful conservation and rational use of plant genetic resources, including both wild and cultivated tetraploid wheat species (Maccaferri et al., 2003; Anuarbek et al., 2020). The appropriate assessment of the genetic diversity in these collections depends on the application of informative and efficient types of DNA markers. In many centers of the world, research is underway to find and use different types of DNA markers with the aim of using them to study genetic diversity, inventory, genotyping, mapping, and identifying genes associated with useful traits of cultivated plant varieties and lines (Idrees, Irshad, 2014). Various types of DNA markers have been developed and are successfully used to study the genetic diversity of accessions of the genus Triticum L. (Röder et al., 1998; Song et al., 2005; Singh et al., 2018). PCR-based markers, such as RAPD, AFLP, and SSR, are widely used tools for studying genetic diversity and discrimination both durum and common wheat (Khlestkina et al., 2002; Kudriavtsev et al., 2004; Yildirim et al., 2011; Abugalieva et al., 2012; Melloul et al., 2014; Adonina et al., 2017).

The wheat genome contains a class of specific nucleotide sequences called microsatellites, also known as SSRs or simple sequences repeats (Ganal, Röder, 2007). SSR markers have many advantages, being highly polymorphic, codominant, informative, reliable, and the availability of information on chromosomal localization (Röder et al., 1998; Vieira et al., 2016). Microsatellites are hypervariable, they often have dozens of alleles at one locus, differing from each other in the number of repeats. They are widely used to study genetic diversity, as well as for the analysis of paternity and mapping of quantitative trait loci (QTLs), kinship, belonging to a specific population, for studying hybridization, evolutionary processes, and for searching for paralogs (Abouzied et al., 2013; Leonova et al., 2013; Jaiswal et al., 2017).

Durum wheat polymorphism studies are currently underway worldwide. The survey of reports demonstrated the successful use of SSR markers for assessment of the genetic diversity in different collections of Europe (Ganeva et al., 2010; Marzario et al., 2018), Africa (Henkrar et al., 2016; Slim et al., 2019), China (Wang et al., 2007; Chen et al., 2012), Russia (Kudryavtsev et al., 2004), Turkey (Yildirim et al., 2011), Syria (Achtar et al., 2010), etc. Microsatellites are also highly effective in tagging specific genes that play an important role in variation for yield components and biotic stress resistance. A number of studies reported relations between SSR loci and wheat traits, such as yield, etc. For instance, Zhang et al. (2013) showed that the Xgwm11-1B locus is significant ( p < 0.001) for plant height. In the study reported by Li et al. (2015) it was shown that the marker Xgwm148-2B is associated with the manifestations of the traits “thousand grain weight”, “spike yield index” and “weight of kernels per spike”. Xgwm251 was associated with lipoxygenase (LOX) activity, which is an important factor determining the color of flour and end-use products of wheat (Geng et al., 2010). Vinod et al. (2014) have identified the significant association between Xgwm234 and the resistance of T. turgidum to leaf rust. Golabadi et al. (2011) showed that the Xcfa2114-6A marker was responsible for 20 % of the phenotypic variation in the yield index and thousand grain weights (TGW) under different environmental conditions. SSR marker Xgwm219 was also shown to be associated with TGW (Roncallo et al., 2017). These examples suggest that the assessment of the genetic diversity of the varietal gene pool of durum wheat may provide not only proper genetic documentation of the accessions but also hinting the identification of a valuable source of genes associated with agronomic traits.

The purpose of this work was the study the genetic diversity using seven SSR markers and phenotypic variation in yield components in the collection of tetraploid species harvested in the conditions of South-East Kazakhstan.

## Materials and methods

Plant material and experimental site conditions. The plant material consisted of 85 accessions of tetraploid wheat (2 Triticum turgidum ssp. dicoccoides (Korn. ex Asch. & Graebn.) Thell., 2 Triticum turgidum ssp. dicoccum (Shrank ex Schübler) Thell., 65 Triticum turgidum L. ssp. turgidum convar. durum (Desf.) MacKey, 10 Triticum turgidum L. ssp. turanicum (Jakubz.) Á. Löve & D. Löve, 4 Triticum turgidum L. ssp. polonicum (L.) Thell., and 2 Triticum turgidum L. ssp. carthlicum (Nevski) Á. Löve & D. Löve from different geographical origins (Supplementary Table 1)^1^. Seeds were provided by the Research Center for Grain and Industrial Crops (Foggia, Italy), University of Bologna (Bologna, Italy), Aktobe and Karabalyk Agricultural Experimental Stations (Kazakhstan). The collection included 21 cultivars and 15 promising lines of durum wheat from Kazakhstan (see Suppl. Table 1).

^1^ Supplementary Tables 1 & 2 are available in the online version of the paper: http://www.bionet.nsc.ru/vogis/download/pict-2020-24/appx9.pdf



The studied collection of tetraploid wheat was evaluated in two randomized replicates in the field conditions of Almaty region (Table 1).

**Table 1.**
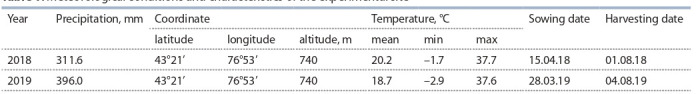
Meteorological conditions and characteristics of the experimental site Note: T mean, T max and T min – average, maximum and minimum temperature during the vegetative period, respectively.

Each accession was planted in two rows with a row spacing of 15 cm, 25 seeds per row. In total, nine agronomic traits connected with the vegetation period, plant morphology, and yield components were studied. The list of traits included the heading time (HT, days), flowering time (FT, days), seed maturation time (SMT, days), plant height (PH, cm), spike length (SL, cm), number of fertile spikes (NFS, pcs), number of kernels per spike (NKS, pcs), thousand kernel weight (TKW, g), and yield per plant (YPP, g) (Anuarbek et al., 2020).

**DNA extraction and SSR genotyping.** Genomic DNA was isolated from individual 4-day-old wheat seedlings, according to Dellaporta et al. (1983). The quality and quantity of isolated DNA were evaluated using a NanoDrop 2000 (Thermo Fisher Scientific, USA) and agarose electrophoresis in 1 % gel. The list of markers used for SSR analysis was the following: Xgwm11, Xgwm148, Xgwm251, Xgwm234, Xcfa2114, Xgwm169, and Xgwm219 (Supplementary Table 2). Polymerase chain reaction (PCR) was conducted in a Veriti™ Thermal Cycler (Thermo Fisher Scientific, USA). The PCR reaction mixture (10 μl) contained from 2.5 mM of 10× Taq buffer; 0.2 mM of each dNTP; 1.5 mM MgCl_2_; 250 μM of each primer; 1 unit Taq polymerase (Promega, USA) and 50 ng of genomic DNA.

The amplification program included the following cycles: 94 °C – 3 min; 40 cycles: 94 °C – 1 min; annealing temperature (55 or 60 °C depending on the primer) – 1 min; 72 °C – 2 min; and 72 °C – 10 min. PCR products were separated on 6 % polyacrylamide gels (Amresco, Solom, OH) run in 0.5× TBE buffer pH 8.0 at 250 V for 1.5 h. Gels were stained with ethidium bromide, and the images were recorded with a Bio- Rad Image System (Bio-Rad, Hercules, CA). Allele sizes were estimated in comparison with 100 bp DNA ladder (Thermo Fisher Scientific, USA).

Statistical analyses of field data were estimated using SPSS 22.0 and STATISTIKA 13.2 software (http://software.dell.com/products/statistica).

Genetic diversity was assessed based on Nei’s genetic diversity index and Shannon Information Index, using the GenAlex, ver.6.5 program (Peakall, Smouse, 2012). The values of the PIC index (polymorphism information content) suggested the effectiveness of the markers used, given that markers with a value of PIC > 0.5 considered as highly informative; 0.5 > PIC > 0.25 as informative; and PIC ≤ 0.25 as marginally informative (Botstein et al., 1980). Variation among populations was studied using Principal Coordinate Analysis (PCoA) in the software GenAlex, ver.6.5 (Peakall, Smouse, 2012). The resulting similarity matrix was further analyzed using the neighbor-joining clustering algorithm for the construction of the dendrogram. The phylogenetic tree was constructed using PAST v.3.25 software (Hammer et al., 2001). Analyses of marker-trait associations were conducted using a simple t-test (Kim, 2015).

## Results

**Phenotypic variation in the studied collection**

Field trials for two years revealed a sharp difference in the vegetation period between species of tetraploid wheat (Table 2).

**Table 2.**
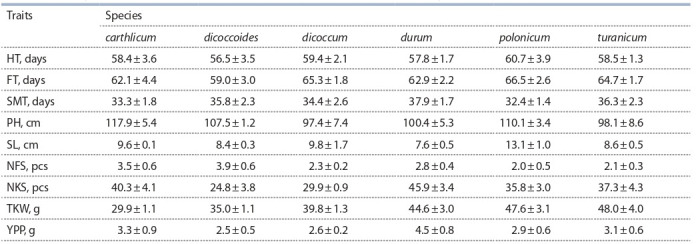
Phenotypic variation in the collection of tetraploid wheat according to two-year field trials data Note: HT – heading time, FT – flowering time, SMT – seed maturation time, PH – plant height, SL – spike length, NFS – number of fertile spikes, NKS – number of kernel per spike, TKW – thousand kernel weight, YPP – yield per plant.

All accessions reached the ripening stage, with an except for the wild accession PI346783 (Hungary, T. dicoccoides). The shortest HT was observed in genotypes of T. dicoccoides (56.5 ± 3.5 days), the longest – in T. polonicum (60.7 ± 3.9 days) (see Table 2).

Plant height is one of the important morphological traits of the crops. According to the species, the highest ones were the samples from T. carthlicum (117.9 ± 5.4 cm), while the accessions from T. dicoccum were the lowest (97.4 ± 7.4 cm). On the other hand for T. durum genotypes the PH ranged from 58.0 ± 3.7 cm (Casanova 58.0 ± 3.7, Mexicali75 58.5 ± 4.9, Ciclope 60.5 ± 3.9) to 137.6 ± 3.0 cm for cultivar Kargala 66 (see Suppl. Table 1). As for the SL, the lowest value (5.0 ± 0.2 cm) had the cultivar PI 184526 (T. turanicum from Portugal), while the highest value (17.5 ± 1.7 cm) was in accession PI 210845 (T. polonicum from Iran).

The value of a cultivar is determined by its productivity, which consists of several components, including TKW which is significantly affected by weather conditions, violation of moisture supply, and mineral nutrition of plants during the formation and maturation of grain. The highest averaged TKW values were revealed for three T. turanicum accessions (CLTR11390, USA – 64.8 ± 4.1 g; PI 352514, Azerbaijan – 58.2 ± 1.0 g; and PI 254206, Iran – 55.2 ± 4.0 g) and T. polonicum from Iraq (PI 208911 – 61.8 ± 4.5 g). The lowest TKW value was in accessions of T. carthlicum (29.9 ± 1.1 g). The NFS ranged from 3.9 ± 0.6 pcs/plant in the accession PI 343446 (T. dicoccoides) to 2.0 ± 0.5 pcs/plant in genotypes PI 210845 and PI 266846 of T. polonicum.

As for NKS and YPP the highest value were on accessions of T. durum and the lowest to T. dicoccoides (see Table 2). The min value of NKS (24.8 ± 3.8 pcs) under both conditions was obtained in PI 343446 (T. dicoccoides, Israel), the max – in Kazakh cultivar Gordeiforme 254 (67.7 ± 7.1 pcs) and Canadian cultivar Strongfield (62.2 ± 1.2). Overall 31 T. durum accessions prevailed the local check cultivar Gordeiforme 254 (4.4 ± 1.6 g/plant) by YPP. Top twenty accessions by yield contained cultivars from Canada (Strongfield – 7.6 ± 1.9 g/ plant), Spain (Granizo – 7.0 ± 1.9 g/plant), Italy (Capeiti-8 and Ancomarzio), Syria (Sharm5), Russia (Har’kovskaya 46, Altaika, Altaiskii yantar’), Ukraine (Har’kovskaya 90 and Har’kovskaya 9), USA (LO92), as well as 5 cultivars and 4 breeding lines (e. g. G 2607 – 7.2 ± 1.4 g/plant), from Kazakhstan (see Suppl. Table 1).

The Pearson index analysis revealed a significant positive correlation ( p < 0.01) between yield components and phenotypic traits. The ANOVA test based on two-years field trials suggested that Genotype significantly influenced the SMT, NFS, SL, and all yield components (NFS, NKS, TKW, YPP) with p < 0.001 (Table 3).

**Table 3.**
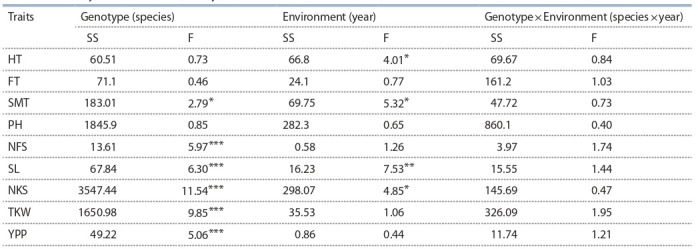
Two-way ANOVA based on two years field trials Note: The F values are provided with significance level indicated by the asterisks. *** p < 0.001, ** p < 0.01, * p < 0.05.

**Microsatellite analysis of the tetraploid wheat collection**

The lines and cultivars of the studied tetraploid wheat collection were analyzed using 7 polymorphic microsatellite markers (see Suppl. Table 2) localized on 6 wheat chromosomes – 1B, 2B, 4B, 5B, 6A, 6B. The results based on using 7 SSR markers have allowed identifying a total of 32 alleles, with average 4.57 alleles per marker (Table 4).

**Table 4.**
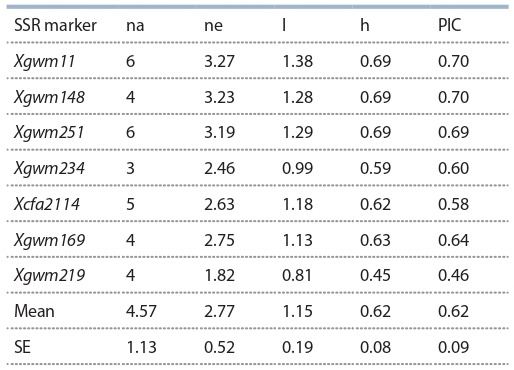
Assessment of the level of genetic diversity of SSR markers in tetraploid wheat collection Note: na – the number of alleles per locus; ne – the effective number of alleles; I – Shannon information index; h – Nei’s diversity index; PIC – polymorphic information content.

The effective number of alleles ranged from 1.82 to 3.27, with a mean value of 2.77. Nei’s genetic diversity index averaged 0.62 (see Table 4). The average value of polymorphism information content (PIC) was 0.62, ranging from 0.46 for Xgwm219 to 0.7 for Xgwm148, Xgwm251, and Xgwm11, respectively.

**Fig. 1.**
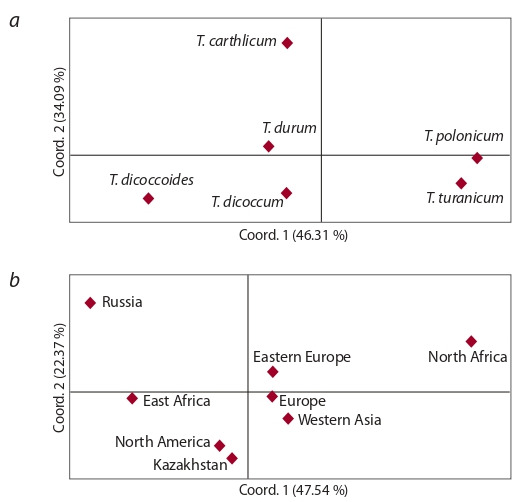
Principal coordinate analysis for 85 tetraploid wheat accessions separated by species (a) and origin (b) groups based on SSR analysis.

The PCoA was conducted based on SSR genotyping of 85 tetraploid wheat accessions using 7 SSR markers. Accessions of the studied collection were divided into groups depending on their attribution to species and place of origin, respectively (Fig. 1).

The first principal component in the PCoA (46.31 %) clearly separated T. polonicum and T. turanicum from other species (see Fig. 1, a). The most genetically distant from other species was T. carthlicum. PCoA using origin data revealed that local genotypes were genetically closer to the North American accessions (see Fig. 1, b). The accessions from Russia and North Africa were genetically distant from other groups of origin.

**Fig. 2.**
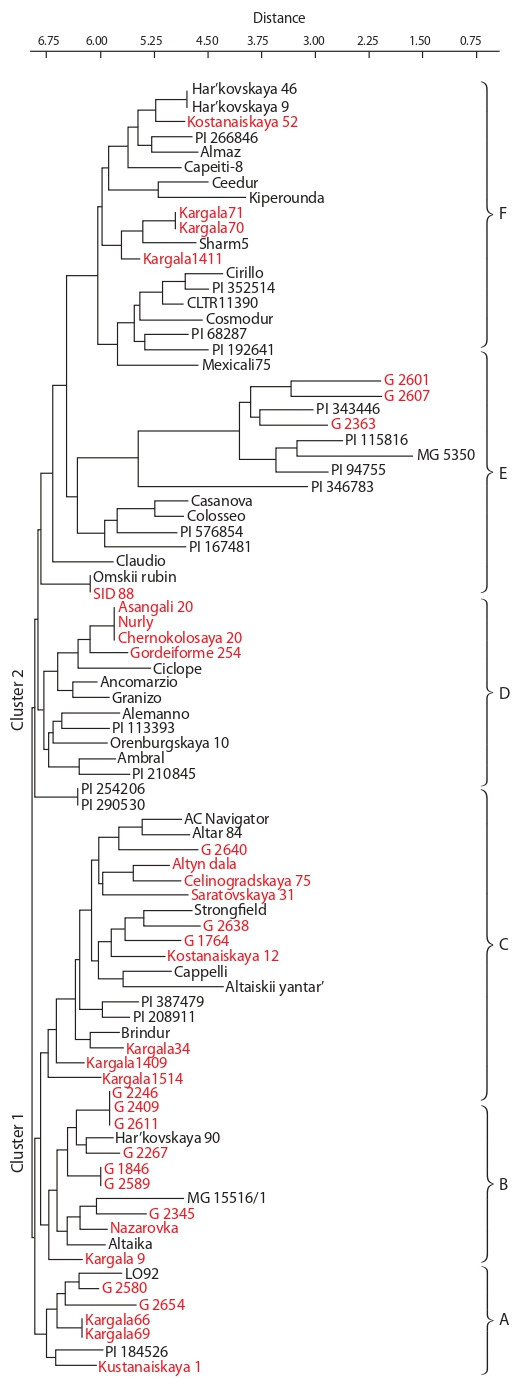
Neighbor-joining phylogenetic tree of 85 tetraploid wheat accessions based on SSR analysis. Sub-clusters are designated as A, B, C, D, E, and F.

Based on the genetic diversity results using 7 polymorphic SSR markers, a phylogenetic tree of 85 accessions of tetraploid wheat was constructed (Fig. 2).

The analysis revealed a division into two large clusters. The first cluster consisted mostly of cultivars of tetraploid wheat from Kazakhstan and North America. The second cluster was divided into three sub-clusters. Although the European accessions were dominated in all three subclusters of cluster 2, all three sub-clusters included cultivars and lines of Kazakhstan (see Fig. 2).

The t-test was performed to confirm the significance of the SSR markers for the studied traits. The results identified the most informative SSR markers related to major agronomic traits (Table 5). Xgwm251 showed a significant relationship to HT and FT. Four markers were related to variance in PH (Xcfa2114, Xgwm251, Xgwm234, and Xgwm169).

## Discussion

Discussion Initially, the studied collection was separated according to their species classification and origin (see Suppl. Table 1). The average yield analysis in the collection of tetraploid accessions over two years (2018 and 2019) suggested that it is highly correlated with all studied phenotypic traits ( p < 0.01), confirming the importance of selected characters in the trials. The two-way ANOVA showed that Environment greatly influenced HT and SMT. In addition, it was found that SMT is also influenced by Genotype, showing the prospects of possibility to adjust maturation time in the breeding process, as early seed maturation is vital to avoid abiotic stresses during the important stages of plant growth. Particularly, it was shown that in T. polonicum the seeds are ripening nearly five days earlier than in T. durum (see Table 2). The field trials have allowed the identification of accessions with outstanding field performances. For instance, the cultivar Strongfield (Canada) showed 7.6 ± 1.9 g/plant, which was the highest yield value among 31 T. durum accessions that prevailed local standard Gordeiforme 254 (4.4 ± 1.6 g/plant). In general, two-way ANOVA indicated the great influence of the environmental factors, as they were affected both adaptation-related traits, such as HT and SMT, and yield components, such as SL and NKS (see Table 3).

The entire collection was studied using seven SSR markers that were located on six different chromosomes (see Suppl. Table 2). According to the previous works, a list of markers in this study was most useful to evaluation of genetic diversity and associations with agronomic traits of durum wheat (Royo et al., 2005). The average PIC value was higher than 0.6, suggesting that the level of polymorphism was very high. The high level of variation in the collection has effectively allowed the separation of accessions according to their species classification (see Fig. 1, a). Notably, the PC1 (46.3 %) separated T. polonicum and T. turanicum from the remaining species, and the PC1 (34.1 %) distinguished T. carthlicum and T. durum from T. dicoccum and T. dicoccoides. Interestingly, the accessions originated in Kazakhstan were genetically close to North American samples (see Fig. 1, b), and it is to some extent confirm the phylogeny of hexaploid bread wheat studies using SNP (single nucleotide polymorphism) markers (Turuspekov et al., 2015). The PC plot is suggesting that six accessions of durum wheat from the Russian Federation are distinctly different from accessions with other origins (see Fig. 1, b). The Neighbor-joining phylogenetic tree suggested that all accessions can be divided into two clusters, where cluster 1 was mostly populated by accessions from Kazakhstan (see Fig. 2).

The significance of each SSR marker for studied traits was assessed using a two-tailed t-test (Lüders et al., 2016; Rahimi et al., 2019). The results of the test suggested that five out of seven SSRs were significant at least for one studied trait (see Table 5). The PH was the trait where four SSR markers, two with negative and two with positive values, were significantly correlated. In addition, the test showed that Xgwm234 is significantly correlated with TKW and Xgwm219 and Xgwm169 with YPP (see Table 5). Thus, the application of SSR markers in the analysis of tetraploid wheat collection consisting of 85 accessions was used for (1) genetic documentation of samples, (2) for phylogenetic clusterization based on the species classification and geographic origin, and (3) associations between DNA markers and studied phylogenetic traits. Hence, the results can be efficiently used for the enhancement of local breeding projects for the improvement of yield productivity in durum wheat.

**Table 5.**
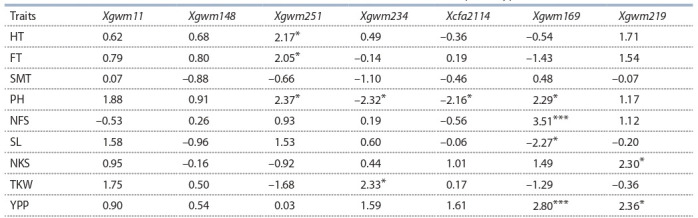
The t-test results on the identification of the relations between SSR markers and phenotypic traits Note: The t-values are provided with significance level indicated by the asterisks. *** p < 0.001, ** p < 0.01, * p < 0.05.

## Conclusion

The phenotypic analysis of the tetraploid wheat collection consisting of 85 accessions showed a high correlation of YPP with all 8 phenotypic traits in conditions of South-East Kazakhstan. The ANOVA suggested that the environmental conditions significantly affected the variation in HT and SMT, while Genotype has contributed significantly to main yield components, including TKW. Overall, 31 accessions of T. durum showed higher average yield values in comparison with local check cultivar Gordeiforme 254 (4.4 ± 1.6 g/plant), and Canadian cultivar Strongfield was with the highest yield value (7.6 ± 1.9 g/plant). The application of seven SSR markers suggested that local accessions were distinctly different from durum accession from other parts of the world. Particularly, the Principal Coordinate plot showed that local durum samples were most close to North American samples. The Neighborjoining phylogenetic tree separated 85 samples to two main clusters, where the cluster 1 was mainly represented by Kazakh accessions and cluster 2 mostly by European accessions. The application of the t-test indicated that five out of seven SSRs were significant at least with one agronomic trait. Obtained results can be efficiently used for the enhancement of local breeding projects for the improvement of yield productivity in durum wheat.

## Conflict of interest

The authors declare no conflict of interest.
